# SDF‐1 inhibits the dedifferentiation of islet β cells in hyperglycaemia by up‐regulating FoxO1 via binding to CXCR4

**DOI:** 10.1111/jcmm.17110

**Published:** 2021-12-21

**Authors:** Xiang‐Yu Chen, Ying‐Xin Shi, Ya‐Ping Huang, Min Ding, Qi‐ling Shen, Chun‐Jun Li, Jing‐Na Lin

**Affiliations:** ^1^ Department of Endocrinology Health Management Center Tianjin Union Medical Center Nankai University Affiliated Hospital Tianjin China; ^2^ Department of Endocrinology and Metabolism Peking University Third Hospital Beijing China; ^3^ Tianjin Medical University Tianjin China; ^4^ NHC Key Laboratory of Hormones and Development, Tianjin Key Laboratory of Metabolic Diseases Chu Hsien‐I Memorial Hospital & Tianjin Institute of Endocrinology Tianjin Medical University Tianjin China

**Keywords:** CXCR4, dedifferentiation, diabetes, FOXO1, islet β cells, SDF‐1

## Abstract

Islet β cell dedifferentiation is one of the most important mechanisms in the occurrence and development of diabetes. We studied the possible effects of chemokine stromal cell‐derived factor‐1 (SDF‐1) in the dedifferentiation of islet β cells. It was noted that the number of dedifferentiated islet β cells and the expression of SDF‐1 in pancreatic tissues significantly increased with diabetes. In islet β cell experiments, inhibition of SDF‐1 expression resulted in an increase in the number of dedifferentiated cells, while overexpression of SDF‐1 resulted in a decrease. This seemed to be contradicted by the effect of diabetes on the expression of SDF‐1 in pancreatic tissue, but it was concluded that this may be related to the loss of SDF‐1 activity. SDF‐1 binds to CXCR4 to form a complex, which activates and phosphorylates AKT, subsequently increases the expression of forkhead box O1 (FOXO1), and inhibits the dedifferentiation of islet β cells. This suggests that SDF‐1 may be a novel target in the treatment of diabetes.

## 1 INTRODUCTION

Diabetes is still one of the most threatening diseases to human health, and is mainly attributed to a low level, or absolute deficiency, of insulin, the principle hypoglycaemic hormone secreted by islet β cells.^1^ Thus, any structural or functional disorder of islet β cells may contribute to diabetes progression. It is generally accepted that all cells originate from embryonic stem cells, through differentiation. However, this process is reversible under certain conditions, including in islet β cells.^2^ Numerous studies have found that islet β cells in a diabetic state can lose their differentiation phenotype, return to the precursor state and lose their insulin secretion function. This is called dedifferentiation and is one of the most important pathogenic processes in diabetes.^3^, ^4^ It is currently unclear why, and how dedifferentiation occurs, and its mechanism is not yet understood.

SDF‐1, also known as CXCL12, is a widely expressed and highly conserved secretory factor, with the homology between humans and mice being as high as 99%.^5^ As a member of the chemokine family, it exerts unpredictable effects on various pathophysiological processes including cell differentiation, immune surveillance and inflammation.^6^ On one hand, SDF‐1 can regulate the differentiation and function of immune cells and play an anti‐inflammatory and immunomodulatory role in type 1 diabetes.^7^, ^8^ On the other hand, a significantly higher plasma SDF‐1 level is common in subjects with type 2 diabetes,^9^ and it is associated with diabetic insulitis, nephropathy and adipose tissue inflammation.^10^, ^11^, ^12^

SDF‐1 is associated with the mediation of islet β cells function. For example, our previous study found that the DPP‐IV inhibitor Saxagliptin improved the function of islet β cells through regulating SDF‐1 expression.^13^ Another study also revealed the important role of SDF‐1 in β cells survival after islet transplantation.^14^ Furthermore, during the terminal differentiation stage of mature β cells, SDF‐1 has been proven to prevent apoptosis and necrosis through activating PI3K/AKT and WNT/β‐catenin pathways.^15^, ^16^, ^17^

Furthermore, SDF‐1 is also related to tissue damage repair in diabetic patients, including heart repair after acute myocardial infarction,^18^ wound vascular healing^19^ and skin scar formation.^20^

In this study, we investigated the effect of SDF‐1 on the dedifferentiation of pancreatic β ‐cells and investigated one of its possible mechanisms through cell experiments based on pancreatic tissues observation. Specifically, active SDF‐1 can bind to CXCR4 and ultimately upregulate FOXO1 expression by phosphorylating AKT, thereby inhibiting the dedifferentiation of pancreatic β cells. However, hyperglycaemia causes a partial loss of SDF‐1activity, which is then unable to bind to CXCR4 or inhibit the dedifferentiation of pancreatic β cells.

## 2 MATERIALS AND METHODS

### 2.1 Synthesis of SDF‐1 siRNA

SDF‐1 small interference RNA was purchased from Gemma gene (item number: Cxcl12‐Mus‐370).

### 2.2 Synthesis of SDF‐1 plasmid

SDF‐1 plasmid was purchased from GeneCopoeia (item number: EX‐Mm05138‐M98).

### 2.3 Reagents

#### 2.3.1 The main antibodies

SDF‐1 (17402–1‐AP, Proteintech, China, RRID: AB_2878404).

SOX9 (ab185966, Abcam, UK, RRID: AB_2728660).

NGN3 (ab216885, Abcam, UK, RRID: Not found).

AKT (10176–2‐AP, Proteintech, China, RRID: AB_2224574).

p‐AKT (ab18785, Abcam, UK, RRID: AB_722674).

α‐tubulin (ab7291, Abcam, UK, RRID: AB_2241126).

Insulin (66198–1‐Ig, Proteintech, China, RRID: AB_2881591).

CXCR4 (11073–2‐AP, Proteintech, China, RRID: AB_2091813).

FOXO1 (18592–1‐AP, Proteintech, China, RRID: AB_10860103).

#### 2.3.2 Others

Goat anti‐rabbit FITC fluorescent secondary antibody kit (BD5003, Bioworld, USA).

Goat anti‐rabbit TRITC fluorescent secondary antibody kit (BD5005, Bioworld, USA).

Goat anti‐mouse TRITC fluorescent secondary antibody kit (BD5004, Bioworld, USA).

DPP‐IV/CD26 (High‐purity dimer) protein (9168‐SE‐010, R&D systems, USA).

Lipofectamine™ 2000 transfection reagent (11668019, ThermoFisher, USA).

Dynabeads™ protein A for immunoprecipitation (10001D, ThermoFisher, USA) Fluorescent mounting tablets (including DAPI) (ZLI‐9557, Zhongshan Golden Bridge, China).

Western primary antibody diluent (ZS402‐1A, ZOMANBIO, China).

4% tissue cell fixative (P1110, Soleibao, China).

Foetal bovine serum (South America) (MP20002‐500 ml, Yuanye Biology, China).

RPMI 1640 medium (C11875500BT, Gibco/Lifetechnologies, USA).

Three‐colour pre‐stained protein marker 10 kDa~250 kDa (WJ103, Epizyme, China), OPTI MEM I (31985062, Gibco, USA).

Four‐colour multi‐labeled immunofluorescence staining kit (abs50012, Absin, China).

4× protein loading buffer (containing β‐mercaptoethanol) (P1016, Soleibao, China) Trypsin‐EDTA digestion solution (PBS) (KGY0012, Nanjing KGI, China).

PAGE gel rapid preparation kit 12.5% (PG113, Epizyme, China).

RIPA lysis solution (strong) (PS0013, Leagene, China).

### 2.4 Human pancreatic tissue specimen

Pancreatic samples were collected from subjects undergoing pancreatectomy caused by non‐neoplastic proliferations. Our study enrolled 3 participants with type 2 diabetes and 3 age‐matched subjects without diabetes, from September 2018 to September 2019. Informed consent forms were obtained from each participant, and the study protocol was confirmed by the Ethics Committee of Tianjin Union Medical Center. The studies were reported in compliance with The Code of Ethics of the World Medical Association (Declaration of Helsinki) for experiments involving humans.

A tissue sample from each subject was immediately fixed in formalin within 240 s after the pancreatectomy, prior to paraffin‐embedding. The remaining specimens were frozen at −80°C for future use. (Ethics number:SZX‐IRB‐ZD‐004(F)‐002–01).

### 2.5 Experimental animal

A total of 15 12‐week‐old male db/db mice (53 ± 4 g) and db/m mice (24 ± 1 g) were purchased from Jiangsu Jicui Yaokang Biotechnology Co., Ltd. Three db/m mice and three db/db mice were randomly selected for the experiment and repeated three times. The mice were housed in an environment at 24 ± 2°C with a 12 h day/night cycle, and had free access to tap water and standard chow diet. The mice were given analgesia immediately before surgery via buprenorphine (0.1 mg/kg−1 intraperitoneally).

All experiments described were approved by the Tianjin Medical University Animal Ethical and Experimental Committee and performed in accordance with their guidelines. Animal studies are reported in compliance with the ARRIVE guidelines and with the recommendations made by the British Journal of Pharmacology.

### 2.6 Cell culture

MIN6 cells were purchased from Beina Biology Co., Ltd. The cells were cultured in 1,640 medium, supplemented with 10% foetal bovine serum and 100 U/ml penicillin and streptomycin in a humidified incubator at 37°C with 5% CO_2_. To construct a dedifferentiation cell model induced by high glucose, different glucose concentrations (11.1 mmol/L, 25 mmol/L, 35 mmol/L, 50 mmol/L and 60 mmol/L) were added to the culture media for 48 h. The optimal concentration based on the dedifferentiation status of the cells was selected. Subsequently, the MIN6 cells were treated for 0 h, 24 h, 36 h, 48 h, 60 h and 72 h, respectively, with the optimal glucose concentration to determine the best intervention time. The optimal glucose concentration and intervention time were then used in the subsequent experiments.

### 2.7 Transfection

#### 2.7.1 Plasmid transfection

Lipo2000 was used for transient transfection (6‐well plate, 4 μg plasmid, 10 μl lipo2000). MIN6 cells were divided into 3 groups: group 1 was supplemented with high glucose, group 2 was supplemented with high glucose and control‐plasmid, and high glucose and SDF‐1‐plasmid were added to group 3.

#### 2.7.2 Small interference RNA transfection

Lipo2000 was used for transient transfection(6‐hole plate; 100 pmol siRNA; 5.0 μl Lipo2000). MIN6 cells were divided into 3 groups: group 1 was supplemented with high glucose, group 2 was supplemented with high glucose and control‐siRNA, and high glucose and SDF‐1‐siRNA were added to group 3.

### 2.8 Tissue immunofluorescence staining

The pancreatic tissue was fixed in 4% paraformaldehyde for 24 h, embedded in paraffin and sectioned into 4 μm slices. Before immunofluorescence staining, the slices were dewaxed in xylene and ethanol, and boiled in 1× TRIS‐EDTA to repair the antigens. Next, the slices were covered with 1%Triton X‐100, diluted by PBS and sealed with 1% BSA diluted by PBS, for 30 min, before being rinsed twice with PBS for 5 min.

#### 2.8.1 Double staining

Insulin (1:800), SDF‐1(1:800) and SOX9 (1:800) antibodies were diluted with primary antibody diluent to incubate the slices overnight at 4°C. The following day, goat‐anti‐rabbit, or goat‐anti‐mouse, fluorescent second antibodies were used to incubate the slices for 1 h at a dilution ratio of 1:400 at room temperature (RT). The slices were then twice washed with PBS for 5 min, covered with DAPI and finally sealed with PBS‐glycerol (1:1). The sections were then observed via a fluorescence inverted microscope.

#### 2.8.2 Triple staining

Triple staining of the pancreatic tissue slices was conducted using a four‐colour multi‐labelled immunofluorescence staining kit. According to the instructions, diluted primary antibody solution was applied on top of the sample area to incubate the tissue at RT for 1 h. The slices were then twice washed with TBST for 3 min. Following that, the tissues were incubated with HRP secondary antibody working solution at RT for 10 min, and the slices were then twice washed with TBST for 3 min. Next, the tissue was covered with 1× 100 ul dye working solution (diluted with a signal magnifier at 100%) at RT for 10 min, and the slices were washed twice with TBST for 3 min. The process was then repeated, and the same slices were stained with different colour immunofluorescence dyes. Finally, the slices were observed via a fluorescence microscope.

### 2.9 Western blot

Equal amounts of total protein extracted from pancreatic tissue or MIN6 cells were set with 12.5% SDS‐PAGE and transferred to a nitrocellulose membrane. After sealing with 5% skimmed milk, the membranes were successively incubated with diluted primary antibodies and HRP‐conjugated secondary antibodies. The primary antibodies included NGN3 (1:1000), SOX9 (1:1000), SDF‐1 (1:1000), CXCR4 (1:1000), α‐Tubulin (1:5000), AKT (1:1000), p‐AKT (1:1000) and FOXO1 (1:1000). Finally, chemiluminescence was detected using an ECL imaging system, and the grey value was analysed via Image J software.

### 2.10 Co‐immunoprecipitation

Firstly, Dynabeads were incubated with a CXCR4 antibody (diluted at 1:50 with primary antibody dilutant) at RT for 10 min and then gently washed with PBS on the magnetic rack for 5 min to remove loose antibodies. Next, protein samples extracted from the cells were added and incubated with the Dynabeads for 10 min. After washing with PBS, the Dynabeads were heated in 1×loading to elute the proteins.

### 2.11 Statistical analysis

GraphPad Prism software (version 8.0) was used for statistical analysis. A Student's *t*‐test was used for comparison between the two groups, and a one‐way ANOVA was used to compare three or more conditions. *p* < 0.05 was considered statistically significant.

## 3 RESULTS

### 3.1 Phenotypic observation of the relationship between SDF‐1 and dedifferentiation of islet β cells

In order to identify the dedifferentiated islet β cells, we selected SRY‐box transcription factor 9 (SOX9). Its specific inactivation in mouse organs can result in exhaustion of the progenitor cell pool, leading to severe pancreatic hypoplasia. SOX9 can also maintain pancreatic progenitor cells by stimulating the proliferation, survival and persistence of pancreatic cancer cells. It can be used as an islet β‐cell dedifferentiation marker,^21^ as when dedifferentiation occurs, its expression level increases. Another dedifferentiation protein marker is Neurogenin 3 (NGN3),^22^ which is highly expressed in pancreatic progenitor cells, and is very important in endocrine differentiation. When dedifferentiation occurs, its expression level increases.

#### 3.1.1 The number of dedifferentiated islet β cells in diabetic patients and diabetic mice is significantly increased

**FIGURE 1:**
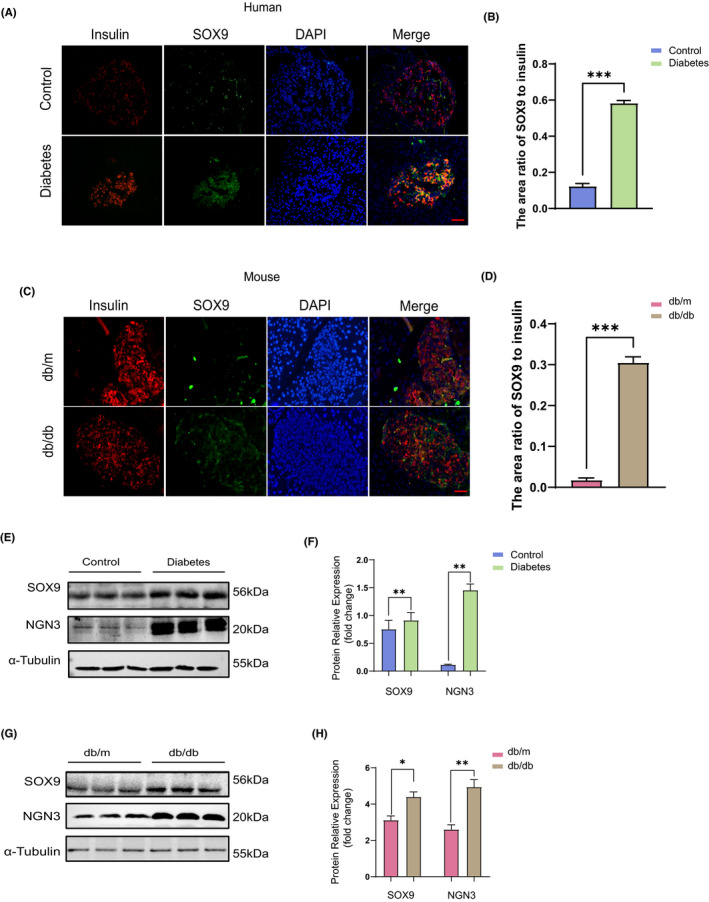
Number of dedifferentiated islet β cells in diabetic patients and diabetic mice is remarkably increased. (A) The paraffin sections of pancreas from patients with and without diabetes mellitus were stained with immunofluorescence double staining, red stands for insulin, green for SOX9 and blue for DAPI. Bar = 100 μm. (B) The area ratio of SOX9 to insulin in islets of control and diabetic patients (*n* = 3). (C) The results of immunofluorescence double staining of paraffin sections of pancreas tissues of db/m and db/db mice, red is Insulin, green is SOX9 and blue is DAPI. Bar = 100 μm. (D) The area ratio of SOX9 to insulin in islets of db/m and db/db mice (*n* = 3). (E) Western blot results and relative expression value results (F) of pancreatic tissue proteins SOX9, NGN3 in control and diabetic patients (*n* = 3). (G) Western blot results and relative expression value results (H) of pancreatic tissue proteins SOX9, NGN3 in db/m and db/db mice (*n* = 3). *p < 0.05,**p < 0.01. ***p < 0.001

In the first set of experiments, we investigated the differences in islet β‐cell dedifferentiation between diabetic and non‐diabetic states. Firstly, paraffin‐treated sections of non‐diabetic and diabetic pancreatic tissues were selected for the double immunofluorescence staining of insulin and SOX9. Red denoted Insulin, green was SOX9, and the insulin‐labelled area is the islet. As can be seen in Figure 1A,B, there was a significantly higher expression of SOX9 in the islets of diabetic patients. In the Western blot, it can be seen that the expression of the dedifferentiation marker proteins SOX9 and NGN3 is increased in the pancreatic tissue of diabetic patients (Figure 1E,F). Thus, it can be considered that more undifferentiated islet β cells appear in diabetic patients. Consistent results were obtained in the db/m and db/db mice experiments (Figure 1C,D,G,H).

#### 3.1.2 SDF‐1 is significantly related to the dedifferentiation of islet β cells

**FIGURE 2:**
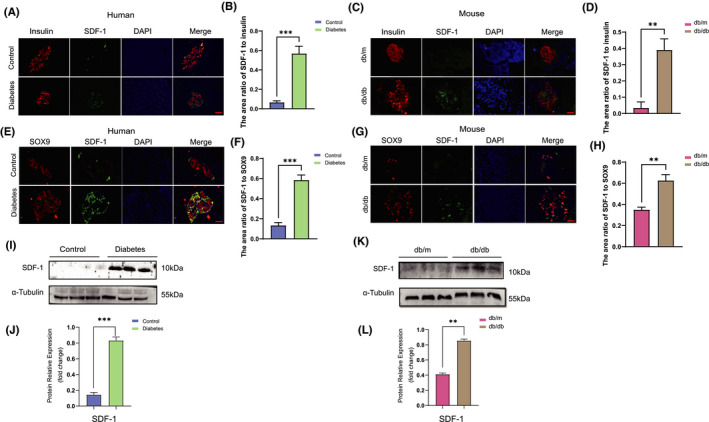
SDF‐1 is significantly related to the dedifferentiation of islet β cells. (A) The paraffin sections of pancreas from patients with and without diabetes mellitus were stained with immunofluorescence double staining, red stands for insulin, green for SDF‐1 and blue for DAPI. Bar = 100 μm. (B) The area ratio of SDF‐1 to insulin in islets of control and diabetic patients (*n* = 3). (C) Paraffin sections of pancreatic tissues of db/m and db/db mice were double‐stained with insulin and SDF‐1 by immunofluorescence. Red stands for insulin, green for SDF‐1 and blue for DAPI. Bar = 100 μm. (D) The area ratio of SDF‐1 to insulin in islets of db/m and db/db mice (*n* = 3). (E) The paraffin sections of pancreatic tissues of diabetic patients and non‐diabetic patients were stained by immunofluorescence double staining, green stands for SDF‐1, red for SOX9 and blue for DAPI. Bar = 100 μm. (F) The area ratio of SDF‐1 to SOX9 in islets of control and diabetic patients. (*n* = 3). (G) The immunofluorescence of paraffin sections of pancreas of db/m and db/db mice showed that red stands for SDF‐1, green for SOX9 and blue for DAPI. Bar = 100 μm. (H) The area ratio of SDF‐1 to SOX9 in islets of db/m and db/db mice (*n* = 3). (I) Pancreatic tissue Western blot results and relative expression value results (J) of non‐diabetic patients and diabetic patients (*n* = 3). (K) The results of Western blot and relative expression value results (L) of pancreatic tissue in db / m and db / db mice (*n* = 3). **p < 0.01,***p < 0.001

Paraffin‐treated sections of pancreatic tissue from diabetic and non‐diabetic patients were double immunofluorescence stained. Insulin labelled with red fluorescence was used to mark the outline of the islet. As shown in Figure 2A,B, the green fluorescence representing SDF‐1 mainly appeared in the islet area and was much denser in the diabetic group. In Figure 2E, SOX9 was used to mark the dedifferentiated islet β cells. Compared with non‐diabetic patients, the expression of SDF‐1 in islet β cells was significantly higher in diabetic patients (Figure 2F), and the fluorescence image was highly overlapped with that of SOX9. Moreover, Western blot also found that the expression of SDF‐1 in the pancreatic tissue of diabetic patients was notably higher than in non‐diabetic patients (Figure 2I,J). Similar results were observed in db/m and db/db mice (Figure 2C,D,G,H,K and L). Thus, it can be deduced that SDF‐1 may be related to islet β cell dedifferentiation.

#### 3.1.3 The expression of SDF‐1 was not significantly changed before or after MIN6 cell dedifferentiation

**FIGURE 3:**
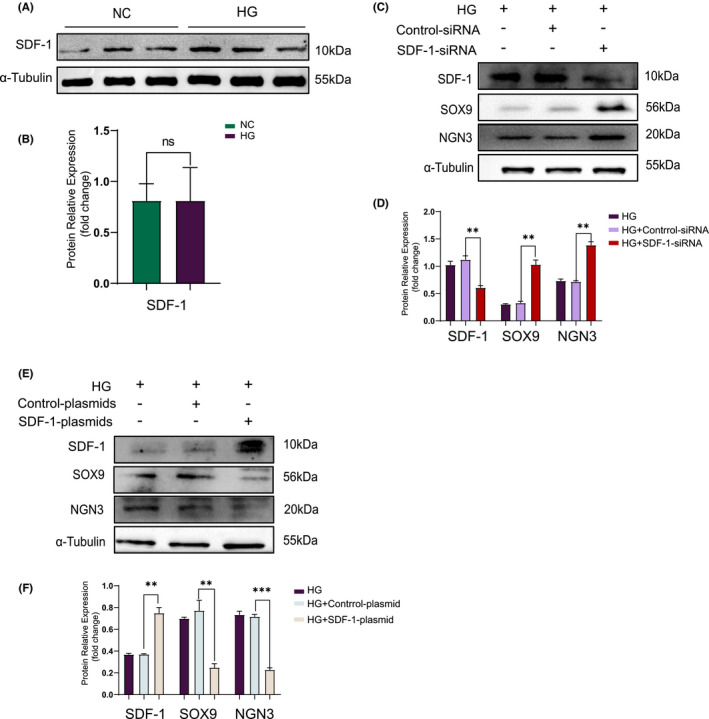
Overexpressing SDF‐1 will decrease the number of dedifferentiated Min6 cells. (A) Western blot and relative expression value (B) of Min6 cells before and after dedifferentiation (*n* = 3). (C) Inhibition of SDF‐1 expression by siRNA in Min6 cells, the results of Western blot and relative expression value results (D) (*n* = 3). (E) After using plasmid to increase the expression of SDF‐1 in Min6 cells, the results of Western blot and relative expression value results (F) (*n* = 3). *p < 0.05, **p < 0.01, ***p < 0.001

We used Min6 cells derived from mouse insulinoma to verify the relationship between SDF‐1 and islet β cell dedifferentiation. Firstly, a dedifferentiation model of Min6 cells was built: Min6 cells were supplemented with different glucose concentrations including 11.1 mmol/L, 25 mmol/L, 35 mmol/L, 50 mmol/L and 60 mmol/L for 48 h, before the cell proteins were extracted for Western blot (Figure S1A). It can be observed that when the glucose concentration was 35 mmol/L, the expression of SOX9 and NGN3 proteins was greatest (Figure S1B), that is, the number of dedifferentiation β cells occurring at this time was the highest. 35 mmol/L glucose was then used to interact with the Min6 cells for 0 h, 24 h, 36 h, 48 h, 60 h and 72 h respectively. Subsequently, the expression levels of SOX9 and NGN3 were detected by Western blot (Figure S1C), and the results showed that the highest value appeared at 60 h (Figure S1D). Therefore, the optimal intervention condition for dedifferentiation of Min6 cells occurred when the glucose concentration was 35 mmol/L with a treatment time of 60 h. However, as shown in Figure 3A,B, under these conditions, there was no significant change in SDF‐1 expression before or after intervention.

### 3.2 Effect and mechanism of SDF‐1 on the dedifferentiation of islet β cells

#### 3.2.1 Overexpression of SDF‐1 caused the number of dedifferentiated Min6 cells to decrease

SDF‐1 expression increased in diabetic pancreatic tissues, but there was no significant change in Min6 cells before or after dedifferentiation, and trends were inconsistent.

Based on the previous tissue experiment results, we originally speculated that reducing SDF‐1 expression would inhibit the dedifferentiation of islet β cells. However, under the preceding Min6 cell dedifferentiation conditions, when transfected with SDF‐1‐siRNA, Western blot results indicated that SOX9 and NGN3 expression was actually upregulated (Figure 3C,D).

Under the same conditions, when enforced express SDF‐1 by transfection with SDF‐1‐plasmid, Western blot results showed that the expression of SOX9 and NGN3 proteins decreased (Figure 3E,F). The above results indicate that decreasing SDF‐1 significantly enhanced the number of dedifferentiated Min6 cells. However, upregulation of SDF‐1 decreased the number of dedifferentiated Min6 cells.

#### 3.2.2 SDF‐1 can phosphorylate AKT, then upregulate the expression of FOXO1 and inhibit the dedifferentiation of islet β cells

FOXO1, a multifunctional transcription factor, plays a complex adaptive role in β cells under metabolic stress.^23^ Inactivation of FOXO1 has been reported in islets of insulin resistance positive GIRKO mice and severely hyperglycaemic db/db mice.^4^, ^24^ In FOXO1‐deficient mice, a large number of islets β cells undergo dedifferentiation, leading to the decrease of functional β cells and further elevated levels of blood glucose.^3^, ^4^

The interaction between SDF‐1 and FOXO1 can be found in the STRING database (Figure S1E). Currently, the most in‐depth study focuses on the SDF‐1/CXCR4 pathway. Therefore, we chose to investigate whether SDF‐1 could affect the dedifferentiation of islet β cells through the SDF‐1/CXCR4/AKT/FOXO1 pathway.

**FIGURE 4:**
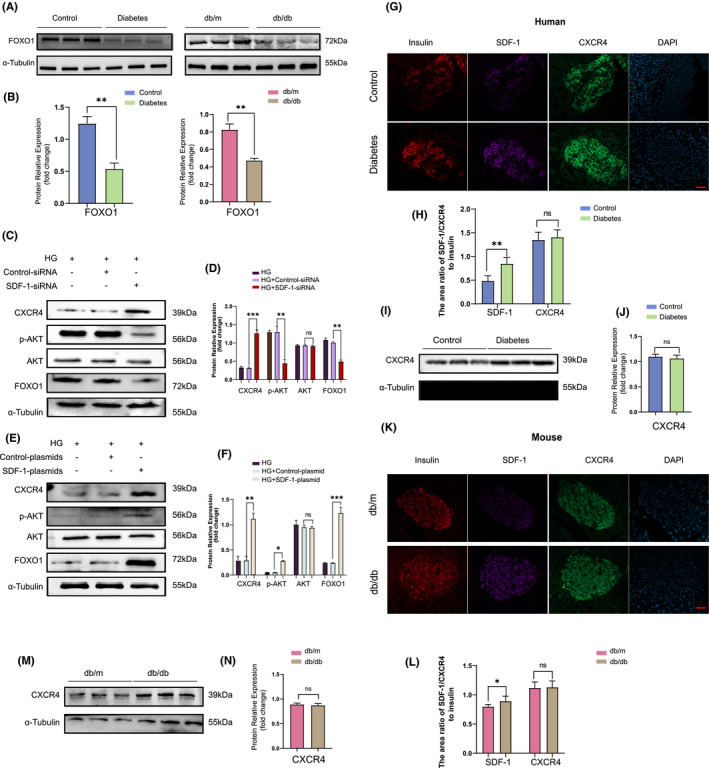
SDF‐1 can phosphorylate AKT, then upregulated the expression of FoxO1 and finally inhibited the dedifferentiation of islet β cells. (A) Western blot results of human and mouse pancreatic tissue proteins under non‐diabetic and diabetic conditions, and the relative expression value results (B) (*n* = 3). (C) In dedifferentiated Min6 cells, the Western blot results and the relative expression value results (D) after inhibiting the expression of SDF‐1 by siRNA transfection (*n* = 3). (E) In dedifferentiated Min6 cells, Western blot results and relative expression value results (F) after plasmid transfection to overexpress SDF‐1 (*n* = 3). (G) Immunofluorescence staining results of pancreatic tissue sections of non‐diabetic patients and diabetic patients. Bar = 100 μm. (H) The area ratio of SDF‐1/CXCR4 to insulin in islets of control and diabetic patients (*n* = 3). (I) Western blot and relative expression value results (J) of pancreatic tissue proteins in non‐diabetic and diabetic patients. (K) The results of immunofluorescence staining of pancreatic tissue sections of db/m and db/db mice. Bar = 100 μm. (L) The area ratio of SDF‐1/CXCR4 to insulin in islets of db/m and db/db mice (*n* = 3). (M) Western blot results and relative expression value results (N) of pancreas tissues of db/m and db/db mice (*n* = 3). *p < 0.005,**p < 0.01,***p < 0.001

To verify this hypothesis, we detected the effect of diabetes on FOXO1 expression in pancreatic tissue via Western blot (Figure 4A). Expression of FOXO1 in pancreatic tissue in a diabetic state was significantly reduced compared to patients/mice without diabetes (Figure 4B). Consequently, we examined the effect of SDF‐1 transfection experiments on the expression of CXCR4, AKT and FOXO1 in Min6 cells. The inhibitory effect of SDF‐1 led CXCR4 expression was augmented, while the levels of p‐AKT and FOXO1 decreased (Figure 4C,D). Enforced SDF‐1 expression resulted in increases in CXCR4, p‐AKT and FOXO1 proteins levels (Figure 4E,F). Therefore, we consider that SDF‐1 can upregulate the expression of FOXO1 and inhibit the dedifferentiation of islet β cells.

However, there are several inconsistencies in the experiments thus far.

1. In the diabetic state, SDF‐1 expression in pancreatic islets and the number of islets β cells undergoing dedifferentiation increase.
2. There was no significant change in SDF‐1 expression before or after Min6 cell dedifferentiation induced by high glucose content.
3. CXCR4 expression is upregulated after the inhibition or rise of SDF‐1 expression in Min6 cells.

To attempt to solve these contradictions, several experiments were conducted.

#### 3.2.3 SDF‐1 inhibited the dedifferentiation of islet β cells after binding with CXCR4 to form a complex

In some cases, SDF‐1 alone did not perform any biological function. However, upon binding with CXCR4 to form a SDF‐1/CXCR4 complex, various effects occur.^25^ Consequently, we explored whether, during the dedifferentiation of islet β cells, SDF‐1 also bound to CXCR4 before it could function.

DPP‐IV can cleave and inactivate SDF‐1, which can then no longer activate CXCR4. In our previous research group studies, we found that compared with non‐diabetic mice, the expression of DPP‐IV in the islets of diabetic mice was enhanced.^13^ Therefore, with a diabetic condition, SDF‐1 was abundantly expressed in pancreatic tissues, but the number of islets β cells undergoing dedifferentiation was not reduced. This suggests that it may be related to the inactivation of SDF‐1 caused by DPP‐IV.

Firstly, we selected pancreatic tissue sections from diabetic and non‐diabetic patients for triple immunofluorescence staining with insulin, SDF‐1 and CXCR4, where insulin marked the extent of the islets (Figure 4G). Although there was a significant difference in SDF‐1 expression between diabetic and non‐diabetic islets, there was no significant difference in CXCR4 expression (Figure 4H). Similarly, no difference in CXCR4 expression between diabetic and non‐diabetic patients was observed in pancreatic tissue Western blot analysis (Figure 4I,J). Consistent results were also obtained in the db/m and db/db mouse pancreatic tissue experiments (Figure 4K,L,M and N).

**FIGURE 5:**
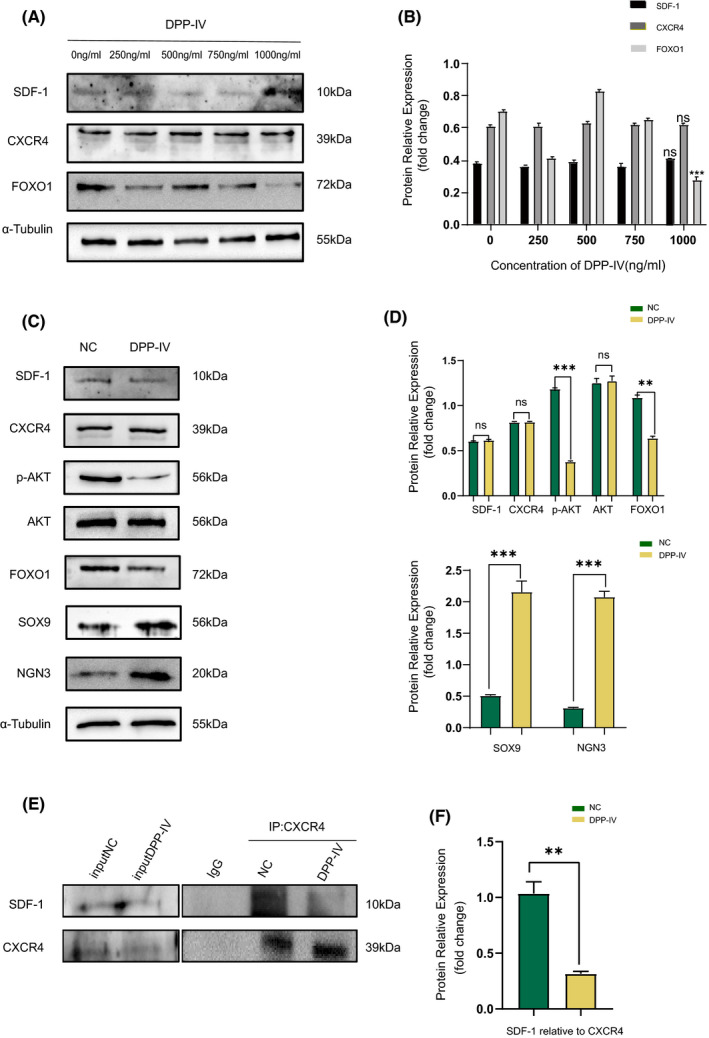
SDF‐1 inhibits the dedifferentiation of islet β cells after it binds to CXCR4 to form a complex. (A) Before and after 48 h of intervention with DPP‐IV of different concentrations in Min6 cells, the results of Western blot and relative expression value results (B) (*n* = 3). ***p < 0.001. (C) Western blot results of Min6 cells before and after intervention with DPP‐IV, and relative expression value results (D) (*n* = 3). (E) The results of CO‐IP and relative expression value results (F) (SDF‐1 relative to CXCR4) before and after DPP‐IV intervention in Min6 cells (*n* = 3). *p < 0.05, **p < 0.01, ***p < 0.001

To investigate whether SDF‐1 affected the dedifferentiation of islet β cells by binding to CXCR4, Min6 cells were treated with different concentrations of DPP‐IV including 250 ng/ml, 500 ng/ml, 750 ng/ml and 1,000 ng/ml for 48 h (Figure 5A). It can be seen that DPP‐IV did not affect the expression of SDF‐1 and CXCR4 but did reduce the expression of FOXO1(Figure 5B). The optimal concentration affecting FOXO1 expression was determined as 1,000 ng/ml; therefore, this intervention concentration was adopted in subsequent experiments.

The Western blot results demonstrated that after DPP‐IV intervention, the expression of SOX9 and NGN3 in Min6 cells was upregulated compared with the non‐intervention group, suggesting that more cells underwent dedifferentiation. At this time, the expression of p‐AKT and FOXO1 was downregulated, while the expressions of SDF‐1 and CXCR4 were not significantly changed (Figure 5C,D). However, CO‐IP results showed that binding of SDF‐1 and CXCR4 was reduced after DPP‐IV intervention (Figure 5E,F).

So, we propose that DPP‐IV did not change the overall expression of SDF‐1 and CXCR4 but did reduce the binding amount between them, which further affected the downstream p‐AKT and FOXO1 pathways, reducing their expression and increasing the number of dedifferentiated β cells.

#### 3.2.4 Verification that SDF‐1 in combination with CXCR4 can upregulate FOXO1expression

**FIGURE 6:**
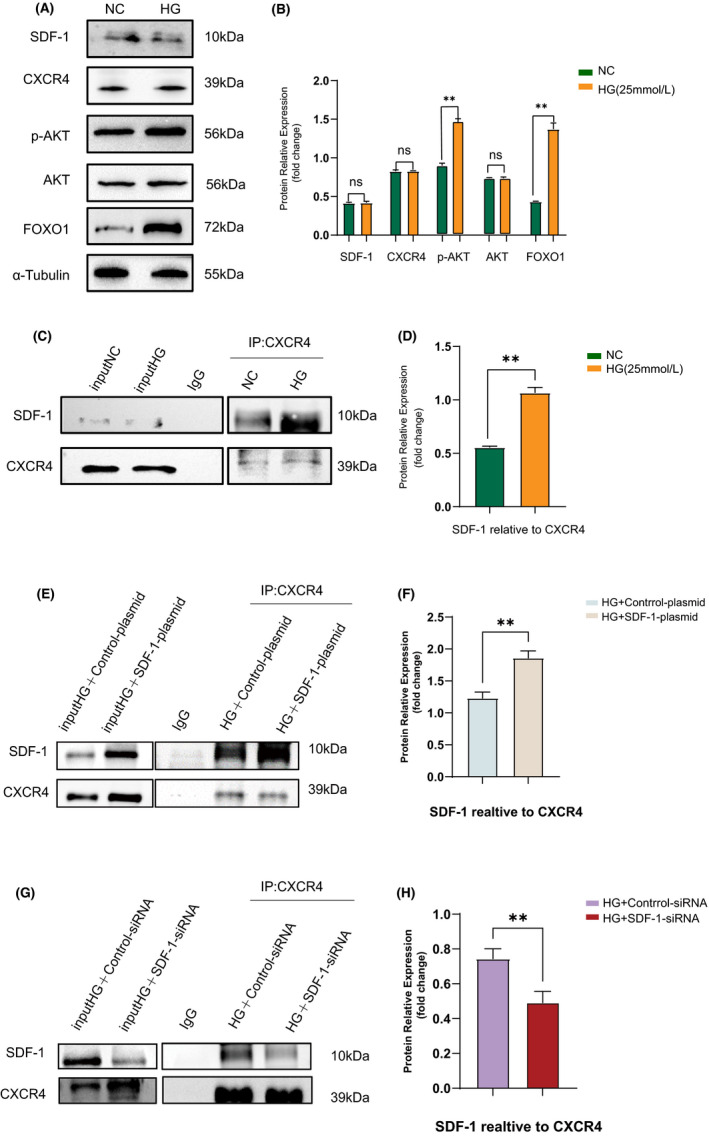
Verify that SDF‐1 in combination with CXCR4 can upregulate the expression of FoxO1. (A) Western blot results and relative expression value results (B) before and after the intervention of 25 mmol/L glucose in Min6 cells for 24 h (*n* = 3). (C) CO‐IP results and relative expression value results (D) (SDF‐1 relative to CXCR4) before and after intervention of 25 mmol/L glucose in Min6 cells (*n* = 3). **p < 0.01. (E) The results of CO‐IP and relative expression value results (F) (SDF‐1 relative to CXCR4) before and after transfection of SDF‐1‐plasmid in Min6 cells (*n* = 3). (G) The results of CO‐IP and relative expression value results (H) (SDF‐1 relative to CXCR4) before and after transfection of SDF‐1‐siRNA in Min6 cells (*n* = 3)

It has been reported that phosphorylated FOXO1 expression in Min6 cells increases after 24 h of 25 mmol/L glucose treatment.^26^ In order to verify this, we examined min6 cells under the same conditions. After glucose intervention, Western blot results showed that the expression of SDF‐1 and CXCR4 had not significantly changed, but the expression of p‐AKT and FOXO1 had increased (Figure 6A,B). The CO‐IP results showed that the relative amount of binding of SDF‐1 to CXCR4 increased (Figure 6C,D). At the same time, we also verified the binding of SDF‐1 and CXCR4 before and after the transfection of SDF‐1‐plasmid and SDF‐1‐siRNA in Min6 cells (Figure 6E,G). Consequently, the binding of SDF‐1 and CXCR4 increased after transfection of SDF‐1‐plasmid (Figure 6F). SDF‐1‐siRNA transfection reduced the binding of SDF‐1 to CXCR4(Figure 6H).

Therefore, we propose that a combination of SDF‐1 and CXCR4 can increase FOXO1expression.

## 4 DISCUSSION

**FIGURE 7:**
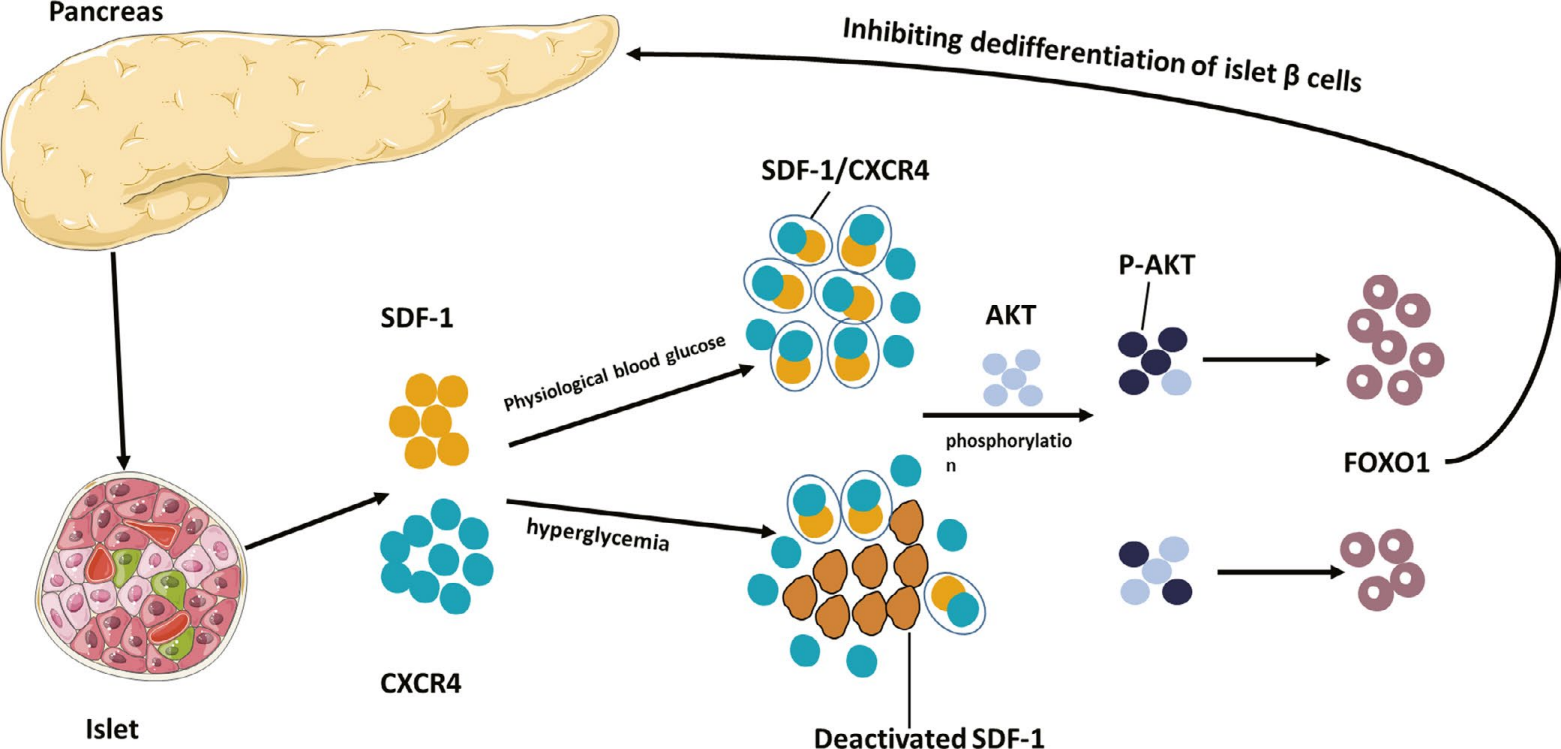
Possible mechanism of SDF‐1 inhibiting the dedifferentiation of islet β cells. Possible mechanism of SDF‐1 inhibiting the dedifferentiation of islet β cells

In this study, we discussed the relationship between SDF‐1 and the dedifferentiation of islet β cells. The results indicated that SDF‐1 can block the dedifferentiation of islet β cells by combining with CXCR4 to form the SDF‐1/CXCR4 complex. This is then followed by phosphorylation of AKT and ultimately by the upregulation of FOXO1expression **(**Figure 7 **)**. However, there were some contradictions in the experiments: (1) Compared with non‐diabetic patients and mice, a higher SDF‐1 expression, and a greater number of dedifferentiated islet β cells, were observed in the pancreatic tissues of diabetic patients and mice. (2) With an increase in dedifferentiated Min6 cells, SDF‐1 expression remained constant, and CXCR4 expression was upregulated, in the transfection experiment.

This may be due to the abundant amount of SDF‐1 expressed in diabetic pancreatic tissues which did not inhibit islet β cell dedifferentiation, and may also be related to the abundant secretion of DPP‐IV after insulin resistance^27^ which inhibited the activity of SDF‐1, reduced the binding of SDF‐1 and CXCR4, and further suppressed the expression of FOXO1. Additionally, although the expression of SDF‐1 did not change before or after the dedifferentiation of Min6 cells, the binding extent of SDF‐1 and CXCR4 was significantly reduced after the dedifferentiation of Min6 cells, which also blocked the expression of FOXO1.

It has been reported that the downregulation of FOXO1 may lead to β cell dysfunction by triggering dedifferentiation and a loss of β cell identity. For example, β cells lacking FOXO1 will re‐differentiate into α cells, displaying high glucagon expression. Under normal glucose conditions, FOXO1 was detected in the cytoplasm of β cells, whereas under mild hyperglycaemia, it was located in the nucleus.^28^ In this research, we observed the downregulation of FOXO1 expression in diabetic pancreatic tissues, when islet β cells were extensively dedifferentiated. Furthermore, we also affected the dedifferentiation of β cells by ‘indirect’ upregulation and downregulation of FOXO1 expression in cell experiments. However, we did not succeed in verifying whether the expression of SDF‐1 affected the nuclear localization of FOXO1.

Islet β cells are plastic, and the number of β cells changes according to the metabolic needs of insulin.^29^ Under certain conditions, dedifferentiated and transdifferentiated β cells can recover their functions. For instance, insulin secretion in diabetic patients and diabetic rats was significantly increased after bariatric surgery.^30^ However, long‐term exposure to hyperglycaemia/stress will cause β cell degeneration and will destroy the plasticity. Some studies have revealed that dedifferentiation occurred before apoptosis.^31^ Therefore, the early recovery of dedifferentiated β cells function is particularly important. We hope that our study on SDF‐1 inhibition of islet β cell dedifferentiation can play a role in the treatment of diabetes.

Islet β cells firstly dedifferentiate into progenitor cells, then transform into glucagon‐producing α cells^32^ and lose their insulin secretion function, which leads to the onset of diabetes. Islet β cell dedifferentiation occurs in both humans and mice.^33^ At this time, functional β cells will be greatly reduced, but not all of them will die. Some of them dedifferentiate into endocrine progenitor cells; others are transdifferentiated into other endocrine cells such as α, δ and Pp.^4^ Consequently, β cells ‘escape from responsibility’ by avoiding excessive metabolic pressure and protect themselves through adaptive mechanisms that avoid cell death under stress conditions.^34^ The dedifferentiation of β cells is a ‘selfish’ protective, and adaptive, mechanism to minimize cell damage.^35^, ^36^ It has been reported that in type 1 diabetic NOD mice, β cell dedifferentiation can be achieved by knocking out the IRE1 α gene, while IRE1α‐deficient NOD mice can be protected from autoimmune damage and the effects of diabetes. Consequently, it remains to be seen whether treating diabetes by inhibiting the dedifferentiation of β cells is only a temporary fix that is bad in the long‐term.

In conclusion, SDF‐1 can inhibit the dedifferentiation of islet β cells, but for a single gene, a single metabolic pathway to inhibit or even reverse dedifferentiated β cells in diabetes treatment, the role of SDF‐1 still has a long way to go.

## CONFLICT OF INTEREST

The authors confirm that there are no conflicts of interest.

## AUTHOR CONTRIBUTIONS

**Xiang‐Yu Chen:** Data curation (equal); Formal analysis (equal); Methodology (equal); Supervision (equal); Validation (equal); Writing – original draft (equal); Writing – review & editing (equal). **Ying‐Xin Shi:** Methodology (equal); Software (equal); Validation (equal); Writing – review & editing (equal). **Ya‐Ping Huang:** Data curation (equal); Validation (equal); Writing – review & editing (equal). **Min Ding:** Conceptualization (equal); Funding acquisition (equal); Project administration (equal). **QI‐Ling Shen:** Formal analysis (equal); Visualization (equal). **Chun‐jun Li:** Conceptualization (equal); Project administration (equal); Writing – review & editing (equal). **Jing‐Na Lin:** Conceptualization (equal); Project administration (equal); Writing – review & editing (equal).

## Supporting information

Fig S1

## Data Availability

The data that support the findings of this study are available from the corresponding author upon reasonable request.
