# Supplementary material for: SDF‐1 inhibits the dedifferentiation of islet β cells in hyperglycaemia by up‐regulating FoxO1 via binding to CXCR4
Source: J Cell Mol Med. 2021 Dec 21;26(3):750–63. doi: 10.1111/jcmm.17110 (PMC8817121; doi:10.1111/jcmm.17110)
Supplement: Supplementary file 1 — Fig S1 [file JCMM-26-750-s001.pdf]

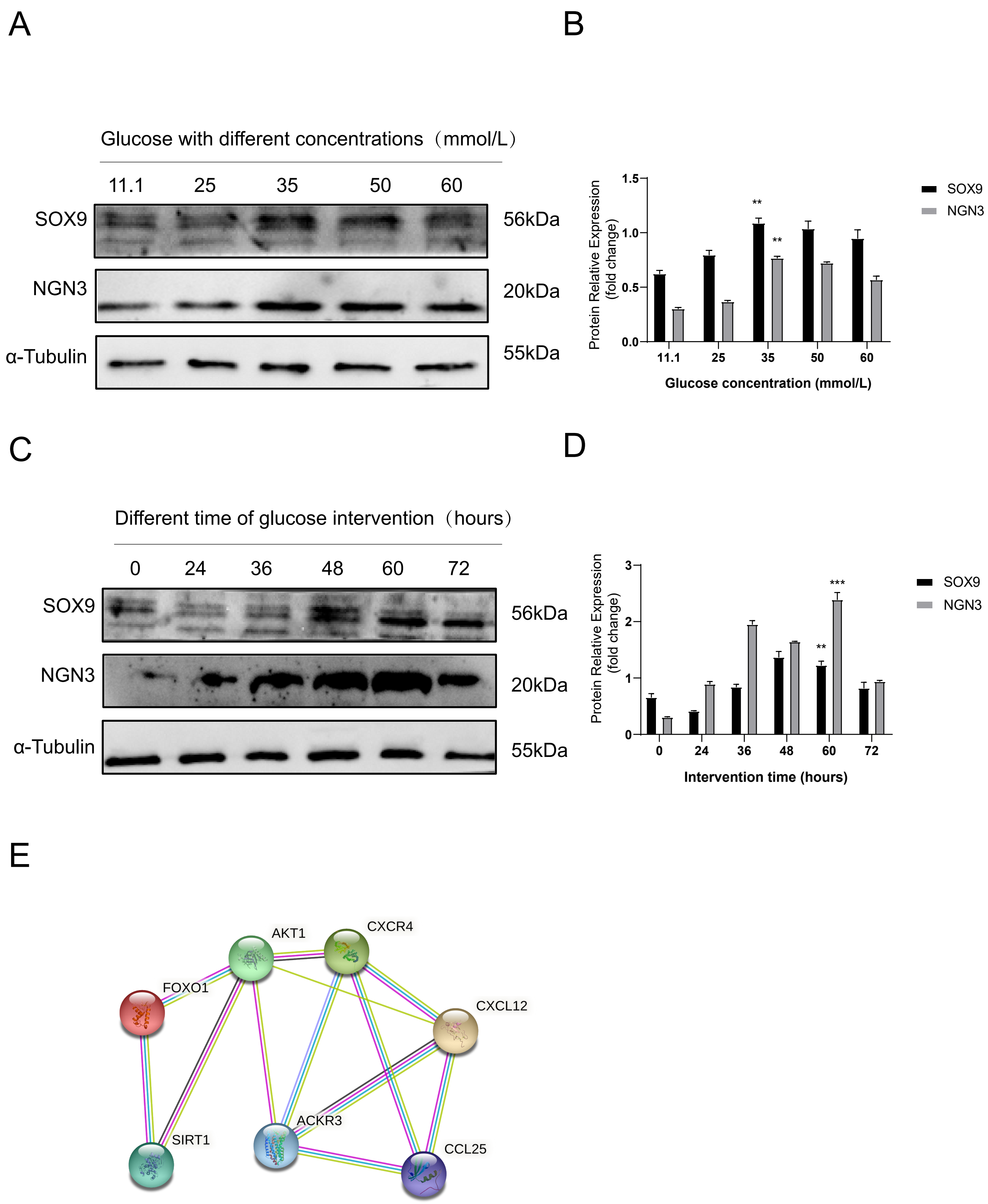
A
Glucose with different concentrations（mmol/L）
11.1
25
35
50
60
SOX9
NGN3
α-Tubulin
56kDa
20kDa
55kDa
B
Protein Relative Expression (fold change)
1.5
1.0
0.5
0.0
**
**
11.1
25
35
50
60
Glucose concentration (mmol/L)
SOX9
NGN3
C
Different time of glucose intervention（hours）
0
24
36
48
60
72
SOX9
NGN3
α-Tubulin
56kDa
20kDa
55kDa
D
Protein Relative Expression (fold change)
3
2
1
0
***
**
0
24
36
48
60
72
Intervention time (hours)
SOX9
NGN3
E
AKT1
CXCR4
FOXO1
CXCL12
ACKR3
SIRT1
CCL25
